# Study protocol: translating and implementing psychosocial interventions in aged home care the lifestyle engagement activity program (LEAP) for life

**DOI:** 10.1186/1471-2318-13-124

**Published:** 2013-11-16

**Authors:** Lee-Fay Low, Jess R Baker, Yun-Hee Jeon, Cameron Camp, Maggie Haertsch, Margaret Skropeta

**Affiliations:** 1 Dementia Collaborative Research Centre, University of New South Wales, Sydney 2052, Australia; 2 Sydney Nursing School, University of Sydney, Camperdown, NSW 2050, Australia; 3 Centre for Applied Research in Dementia, 34208 Aurora Road, #182, Solon, OH 44139, USA; 4 Arts Health Institute, PO Box 1772, Newcastle, NSW 2300, Australia; 5 School of Science and Health, University of Western Sydney, Campbelltown Campus, NSW, Australia

**Keywords:** Home care, Psychosocial intervention, Engagement, Ethnic minority

## Abstract

**Background:**

Tailored psychosocial activity-based interventions have been shown to improve mood, behaviour and quality of life for nursing home residents. Occupational therapist delivered activity programs have shown benefits when delivered in home care settings for people with dementia. The primary aim of this study is to evaluate the effect of LEAP (Lifestyle Engagement Activity Program) for Life, a training and practice change program on the engagement of home care clients by care workers. Secondary aims are to evaluate the impact of the program on changes in client mood and behaviour.

**Methods/design:**

The 12 month LEAP program has three components: 1) engaging site management and care staff in the program; 2) employing a LEAP champion one day a week to support program activities; 3) delivering an evidence-based training program to care staff. Specifically, case managers will be trained and supported to set meaningful social or recreational goals with clients and incorporate these into care plans. Care workers will be trained in and encouraged to practise good communication, promote client independence and choice, and tailor meaningful activities using Montessori principles, reminiscence, music, physical activity and play. LEAP Champions will be given information about theories of organisational change and trained in interpersonal skills required for their role. LEAP will be evaluated in five home care sites including two that service ethnic minority groups. A quasi experimental design will be used with evaluation data collected four times: 6-months prior to program commencement; at the start of the program; and then after 6 and 12 months. Mixed effect models will enable comparison of change in outcomes for the periods before and during the program. The primary outcome measure is client engagement. Secondary outcomes for clients are satisfaction with care, dysphoria/depression, loneliness, apathy and agitation; and work satisfaction for care workers. A process evaluation will also be undertaken.

**Discussion:**

LEAP for Life may prove a cost-effective way to improve client engagement and other outcomes in the community setting.

**Trial registration:**

Australian New Zealand Clinical Trials Registry ACTRN12612001064897.

## Background

Aged care will increasingly be provided at home rather than in institutions [1,2]. This trend is driven by the wishes of older people to stay at home [3-6], and changing social structures reducing the availability of informal family care. Although desirable, there are negative aspects to living at home as an older person. A third of community dwelling older persons have reported being lonely, most of these (80%) did not receive home visitation or telephone contact services, and 40% did not attend any group activities [7]. Community dwelling older persons have also reported having limited access to meaningful activities [8]. Older people who receive home care (also known as community or domiciliary care) services want personalised activities and opportunities for socialisation [3]. The most frequent unmet needs of persons with dementia living alone were daytime activities (54%), company (52%), and care for psychological distress (44%) [9,10]. Home care recipients’ ratings of their opportunities for activities were significantly lower than residents in nursing homes [11]. Forty-two per cent of Australians referred to home care screened positive for depression [12], and similarly 43% of American home care clients met DSM-IV criteria for depression [13]. Sixty per cent of community dwelling persons with dementia have behavioural disturbances [14]. These behaviours such as agitation, aggression, psychosis, anxiety and depression are stressful and increase the burden of carers, and increase the likelihood of institutionalisation [15].

Psychosocial activity-based interventions, particularly tailored activities, have been shown to improve outcomes for residents in aged care facilities and are recommended in many dementia management guidelines [16,17]. For instance, music and diversional therapy decrease depression, anxiety and social withdrawal for persons with dementia [18]. Aromatherapy, preferred music, muscle relaxation training, Montessori activities and humour therapy have been shown to reduce behavioural disturbances in agitation, aggression and verbal disruption [19-21]. Individualised activity programs tailored to older person’s cognitive, physical, and sensory abilities, and their lifelong habits and roles have been particularly effective in decreasing agitation, depression and improving quality of life of persons with dementia [22]. Common to these interventions is engagement of clients, which may fulfil unmet needs for company or meaningful activity and stimulation [23]. We believe that engagement of residents through activity is the critical ingredient for these interventions.

Only a few tailored psychosocial activity programs have been trialled in community settings for people with dementia [24,25]. All involved use of experienced therapists in addition to existing care. A 10 session in-home program conducted by occupational therapists included environmental modification, and training for patients and carers on how to optimise functional performance. This program reported improving the mood and quality of life of persons with dementia [25]. The Tailored Activity Program (TAP) showed that assessment informing tailored activity prescription by an occupational therapist coupled with caregiver training over eight sessions at home reduced problem behaviours and increased pleasure and engagement of persons with dementia [24]. TAP costs $961.63 per caregiver-client dyad and sustainability of effects is not known as there was no follow-up beyond four months [25].

The aim of this study is to evaluate the effects of LEAP for life on increasing home care client engagement by aged care staff. The effects of the program on client mood, behaviour and satisfaction with care, and care worker satisfaction with work will also be measured.

## Methods/design

The design of the program was informed by Greenhalgh’s framework for diffusion of innovations in service organisations [26] and the literature on implementation and culture change in health and aged care [27-35]. Cost, scalability and sustainability were also considerations of the program design. We did not specifically base the program design on a theory, as there was no published theory that appeared suitable with a sound evidence base in predicting good implementation outcomes [36]. The three components of the program are:

1) Engaging site management and care staff with the LEAP for Life program

2) Employing a LEAP champion one day a week at each site

3) A training program (detailed below)

**Figure 1:**
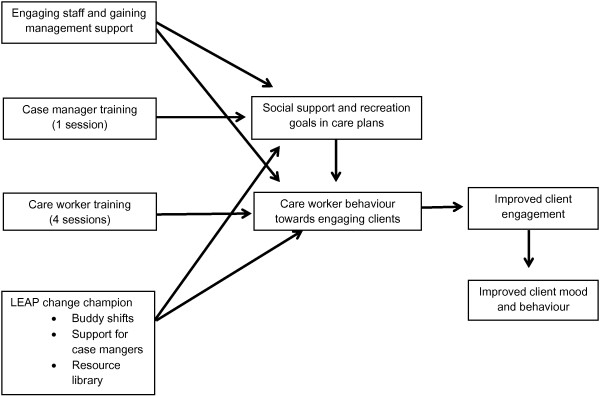
LEAP for life program logic model.

The program logic model of the implementation is outlined in Figure 1. LEAP will encourage case managers to include social and recreational goals in client care plans; and care workers to increase behaviours which engage clients socially and recreationally. Behavioural change of care workers and case managers is to be driven by a LEAP champion within each site. By increasing client engagement, we hypothesise that client depression, loneliness, apathy and/or agitation will improve for those with symptoms in these areas, and that satisfaction with care will also increase. We also hypothesise that relationships between care staff and clients, and care staff work satisfaction will increase.

A position description has been written for the LEAP champion and champions will be selected after discussion with site managers. The role of the LEAP champion is to: support care workers in ways to meaningfully engage clients; assist case managers in including social or recreational goals within clients’ care plans; develop personalised resources for client engagement; develop an activities library; accompany care workers on buddy shifts; and to liaise with the research team.

### Development of the training sessions

The training sessions have been developed based on literature reviews and consultation with experts as detailed under each training component below. We also reviewed the literature on how to best deliver training for aged care workers [37-39], and drew on the experience of the Advisory and Steering committee. The training program was designed around the principles that it was to be interactive, practical and skills based; allow care staff to experience the impact of the psychosocial strategy; include self-reflection and discussion; acknowledge care staffs’ expertise and experiences; take a problem solving approach; inspire; and be fun. The training program was also developed to cater for staff with different levels of education, English language proficiency and cultural backgrounds [40].

Barriers to implementing lifestyle activities in home care were identified through the advisory and steering groups and from -6 month interviews with case managers at participating sites. Barriers identified were: lack of time, lack of knowledge, lack of resources, and attitudes of families and clients that the remit of the care worker is to just provide practical or physical assistance. These have been explicitly addressed in the training and practice change program.

The training program structure is outlined in Table 1. Each training session was refined using feedback from the advisory group, steering committee, and following a pilot session at a non-participating service.

**Table 1:** LEAP for life training program lesson topics, learning objectives, activities and duration

**Training lesson topic**	**Learning objectives**	**Training activities**	**Time (hours)**
Case Manager			
Introduction to LEAP and goal setting with clients	• To understand why the LEAP for Life program is important	1. Introduction of facilitator/s and ground rules	3
	• To learn SMART social and recreational goal setting skills	2. Icebreaker –where key life moments are shared to emphasise the importance of socialisation and recreation	
	• To learn how to select and modify goals for community aged care clients in relation to their cognitive and functional ability	3. LEAP for Life within a community aged care context: a) Maslow’s hierarchy of needs b) the importance of LEAP b) how to implement LEAP in community aged care	
	• To learn how to incorporate social and leisure goals into current care plans with community aged care clients	4. Workshop as a group a) setting a SMART goal, and b) setting SMART goal sub-steps	
	• To explore ways of and barriers to, implementing the skills and strategies learnt in training, into their usual practice	5. Role play in pairs setting SMART goals with clients	
		6. Brainstorm as a group how to maximise implementation and sustainability of LEAP within own organisation	
		7. Self-reflection activity on what each person will put into practice from the session	
Care worker			
Introduction to LEAP, communication skills, engaging clients and reminiscence	• To understand the importance of engaging and supporting clients through socialisation and recreation	1. Introduction of facilitator/s and ground rules	3
	• To understand the purpose of the LEAP for life program, and program structure	2. “Whoosh” ball game icebreaker: a physical group activity to get the group engaged, comfortable and having fun	
	• To understand that goal setting with clients will occur, and how they may contribute to achieving goals	3. Brainstorm as a group the care workers role and what care workers like/dislike about their job	
	• To understand how to involve and engage clients more during usual care	4. Overview of LEAP for Life: a) the program logic model b) an understanding that goal setting with clients will occur	
	• To improve listening skills using body language and questioning	5. Brainstorm as a group the benefits of keeping active	
	• To understand the principles of reminiscence	6. Brainstorm as group how to involve clients more during usual care activities	
		7. Communication skills pair work exercises: a) the importance of good body language b) active listening – paraphrasing, reflecting, mirroring, paying compliments	
		8. Brainstorm as a group how to get people talking and play “six degrees of separation” game in pairs.	
		9. Brainstorm as a group how to encourage clients to reminisce	
		10. Group discussion about what to do when the conversation goes wrong	
		11. Self-reflection activity on what each person will put into practice from the session	
Engaging clients with dementia	• To revise previous session behavioural and psychological symptoms of dementiaTo understand the	1. Introduction of facilitator/s and ground rules	2
	• To develop skills on communicating with people with dementia	2. Group discussion about strategies learnt or practiced last session	
	• To develop skills on task analysis	3. “Whoosh” ball game icebreaker: a physical group activity to get the group engaged, comfortable and having fun	
	• To develop skills in using Montessori principles to present and tailor activities for people with dementia	4. Dementia: a) an understanding that people with dementia may express challenging behaviour because of an unmet need or changes in the brain b) an understanding that LEAP for Life may help to address unmet needs	
		5. Brainstorm as a group good practices when communicating with people with dementia	
		6. Broken Telephone activity to demonstrate the importance of clear and slow speech and “looking” for feedback	
		7. Balloon modelling mirroring activity to demonstrate the value of modelling rather than describing a task	
		8. Brainstorm as a group task analysis and modification of making a cup of instant coffee	
		9. Montessori Principles: a) introduction, b) sorting activity demonstration with cutlery c) group brainstorm as to other activities one might be able to do around the house with a client with dementia d) role play in pairs turning a household object into an activity with a person with dementia	
		10. Self-reflection activity on what each person will put into practice from the session	
Incorporating music and physical activity into daily care	• To revise previous session	1. Introduction of facilitator/s and ground rules	2
	• To understand and experience the benefits of music	2. Group discussion about strategies learnt or practiced last session.	
	• To learn how to select and incorporate into daily care individualised music for clients	3. “Name that song” group activity to experience the enjoyment and reminiscence effects of music	
	• To learn how to encourage a person with dementia to actively engage with music	4. Brainstorm as a group how to select music with and for clients and for different moods	
	• To understand the benefits of physical activity	5. Brainstorm as a group how to incorporate music into daily care with clients	
	• To learn how to incorporate physical activity into daily care	6. Group singing activity to give care workers confidence in singing and to experience the benefits of engaging in music with others	
	• To learn about activities and approaches that might be most suited to engaging male clients	7. “Follow my leader” paired activity to practice encouraging people with dementia to move along to music	
		8. Brainstorm as a group activities and approaches most suited to engaging male clients	
		9. Facts about exercise trivia quiz to emphasise benefits of physical activity	
		10. Brainstorm as a group how to encourage clients to be physically active	
		11. Self-reflection activity on what each person will put into practice from the session	
Humour and play and status and reciprocity	• To revise previous session	1. Introduction of facilitator/s and ground rules	2
	• To understand what play and humour are and their benefits	2. Group discussion about strategies learnt or practiced last session	
	• To understand appropriate and inappropriate play and humour	3. Chair swap circle game: a physical group activity icebreaker to get the group engaged, comfortable and having fun; and to experience the playful approach important in initiating a playful encounter	
	• To develop skills to be more playful with clients	4. Group discussion about what play is, and the difference between a playful activity and a playful state	
	• To develop problem-solving skills and creativity	5. Brainstorm as a group the social, emotional and physical benefits of play	
	• To explore barriers to play and humour with clients	6. Group discussion about using play at work focused on promoting the understanding that being playful at work will increase creativity, and in turn improve problem-solving ability	
	• To understand and develop strategies for increasing the status of clients	7. Circle drawing activity to practice thinking creatively	
	• To identify practices to maximise sustainability of LEAP within organisation	8. Group problem-solving exercise about a hypothetical challenging client to practice applying creative thinking to work problems	
		9. Group discussion about Marin’s four distinct styles of humour, and self-reflection on own humour style	
		10. Brainstorm as a group ways to be more playful and humorous with clients	
		11. Brainstorm as a group risks and barriers of being playful with clients	
		12. Group discussion about the concept of status and the loss of status in older people	
		13. Brainstorm as a group ways to increase the perceived status of clients	
		14. Group reflection about the skills care workers have learnt over the course of the LEAP training program	
		15. Brainstorm as a group ways in which the care organisation can maximise the sustainability of LEAP	
LEAP Champion			
The LEAP Champion role, organizational change and interpersonal skill sin person persuasion	• To get to know other LEAP champions and form a collaborative network;	1. Introduction of facilitator/s and ground rules	5
	• To be clear about LEAP champion role within own organisation;	2. Icebreaker – getting to know the other LEAP Champions and finding out three things each has in common	
	• To anticipate difficulties in promoting change within organisation and brainstorm solutions;	3. Brainstorm as a group the LEAP Champion role	
	• To improve interpersonal skills in person persuasion – active listening, constructive feedback, negotiation, assertiveness, and persuasion;	4. The change process: a) Roger’s diffusion of innovations process, b) Roger’s adopter categories	
	• To define how LEAP will work in each individual’s own schedule and organisation.	5. Brainstorm as a group what characteristics and skills the LEAP Champion will ideally have	
		6. Self-reflection exercise on each individual’s strengths and challenges in regards to the LEAP Champion role	
		7. Communication and Influencing Skills: a) good listening pair work exercise b) giving feedback – pair work exercise using the sandwich technique c) assertiveness skills group role-play d) negotiation tactics and persuasion tips e) persuasion skills pair work exercise	
		8. Groups discussion about the buddy shift	
		9. Individual exercise planning how each LEAP champion will manage their one work day of LEAP	
		10. Brainstorm as a group how UNSW will support the LEAP champions in their role	
		11. Self-reflection activity on what each person will put into practice from the session	

### LEAP champion training

One five-hour champion training session informed by the literature [41-44], will be delivered three months prior to the start of the program. The training will clarify the LEAP champion role including specific tasks to be performed, educate LEAP champions in the process of change and responses to change; develop interpersonal skills for person persuasion; identify potential difficulties and brainstorm solutions within their sites; and encourage networking between the LEAP champions.

### Case manager training

One three-hour training session program informed by the literature and consultation with experts [45-48], will be delivered at the beginning of the program. The training introduces case managers to the aim and importance or need for LEAP and trains them in setting SMART (Specific; Measurable; Achievable; Relevant: Timely) goals [49]. The training will conclude with a brain storming session on specific strategies that could be used to fully implement LEAP within their organisation. Strategies will be fed back to each site to review, collectively finalise and action.

### Care worker training

Care workers will participate in four two or three hour training sessions, held every three months. The staggered sessions will allow time to apply and consolidate sessions and act as boosters to continue using the principles of engagement taught in LEAP. Training has been designed to be delivered on-site to small groups of between 6–10 case managers or 10–20 care workers.

Care worker training was developed based on the following principles and psychosocial strategies for which there was an evidence base in improving outcomes: taking a person-centred individualised approach [50], dementia and the unmet needs model of challenging behaviours [23], communication skills [51], autonomy and control [52,53], reminiscence [54,55], music [56], physical activity [57], Montessori activities [58] humour [59] and reciprocity [60].

Over the course of the training program, the LEAP Champion will also accompany each care worker on a buddy visit to a client in order to support care workers in practising client engagement techniques. Training materials will be translated into Chinese and delivered by a trilingual trainer (English, Mandarin, and Cantonese) in a Chinese speaking site.

### Inducting new staff members

Staff turnover in home care is high [40]. LEAP champions will review and discuss LEAP for Life with care workers who missed training sessions, using a provided script and handout of key points. New case managers will also attend a brief training session.

### Evaluation

The study has been approved by the University of New South Wales Human Research Ethics Committee (HC12383) and registered on the Australian New Zealand Clinical Trials Registry (ACTRN12612001064897).

### Setting

The project will be evaluated with five aged care community service providers in regional and metropolitan New South Wales, Australia. The providers were approached to participate by the chief investigator. Two of the sites specifically provide services for clients from ethnic minority, non-English speaking backgrounds. These services deliver case managed home care packages. The types of services provided by these packages include personal care, domestic assistance, social support, travel to appointments, in-home respite and nursing care.

Eligibility criteria for participating services are:

• Government accredited, providing home care packages to older people in the community;

• Not enrolled in another intervention study relating to engagement and activities;

• Willing to sign a legal contract with regards to program terms.

### Study participants and recruitment processes

1) *Case managers and care workers*

Site managers or their nominees will invite all staff members working on home care packages to participate in the study and provide them with a study flyer, information sheet and consent form. Care workers will either be paid for their time participating in the evaluation or receive a small inducement to complete questionnaires. Based on discussions with site managers, we anticipate that all of the 20 case managers and 90% of the 183 care workers (i.e. 165) will consent to participate.

2) *Community aged care clients*

All eligible clients (*n* = approximately 422) being cared for by the service under a home care package will be invited to participate. A 50% participation rate and 25% attrition is anticipated, thus about 211 clients will be recruited, and 158 clients will provide complete client data over 18 months. Clients will be ineligible if they are; foreshadowed to stop accessing the service, acutely unwell, or under public guardianship with no person responsible to consent on their behalf.

Case managers or their nominee will invite all eligible clients to participate in the evaluation by briefly introducing the study and giving them and/or their families a study flyer. Clients and their families will give verbal assent to case managers to have their details passed onto the research team who will explain the study and provide a written information and consent form. Cognitively intact clients and families will each provide written consent for their own participation. When the client may be cognitively impaired, verbal assent will be obtained from the client, and family written consent will be obtained for both family and client participation.

### Design

LEAP is being evaluated using a quasi-experimental design with measurements taken at -6, 0 (baseline immediately pre-intervention), 6 and 12 months. The time period from -6 to 0 months will act as non-intervention comparison for the period between 0, 6 and 12 months. Clients will be clustered under their case managers (*n* = 20).

#### Assessment tools

##### Primary outcome measure: client engagement

Client engagement will be assessed from both the care worker and client perspective. A six-item purpose-specific care worker questionnaire will measure the five dimensions of engagement conceptualised in Cohen-Mansfield’s Observational Measure of Engagement (OME) - for which there has been demonstrated sound inter-rater reliability and construct validity [61]. The five dimensions are: client rate of refusal of offers of interaction; proportion of time during care worker visits that the client is involved with an activity or conversation; client attention level; client attitude towards any interaction; and the appropriateness of client interaction. In order to ascertain test-retest reliability of the measure, 20 care workers will be asked to complete the questionnaire twice, two weeks apart.

Client perspectives on engagement with care staff will be rated by researchers after conducting semi-structured interviews with the clients and families. Researchers will rate four engagement items pertaining to frequency of and feelings towards social conversation or recreational activities during care worker visits. A random selection of 10 interviews shall be scored by a two researchers, in order to establish inter-rater reliability.

##### Secondary outcome measures

Secondary outcome assessment tools for both client and care worker are detailed in Table 2.

**Table 2:** Secondary outcome measures for client and care staff and the covariates

	**Measure**	**Data collection method**
Client secondary outcome		
Agitation	34-item Cohen-Mansfield Agitation Inventory-relative[62]	Family self-report
	13-item Agitation subscale of The Neuropsychiatric Inventory – Clinician Rating Scale [63]*	Client/family interview
Dysphoria/depression	15-item Geriatric Depression Scale [64]^†^	Client self-report
	13-item Dysphoria subscale of The Neuropsychiatric Inventory- Clinician Rating Scale [63]*	Client/family interview
Loneliness	20-item Revised UCLA Loneliness Scale [65]‡	Client self-report
Apathy	18-item Apathy Evaluation Scale – self and informant [66]	Client and family self-reports
	11-item Apathy subscale of The Neuropsychiatric Inventory - Clinician Rating Scale [63]*	Client/family interview
Satisfaction with care	9-item care worker subscale and 13-item case manager subscale of the Home Care Satisfaction Measure [67]	Client self-report
Client Covariates	age; gender; care site; hours and type (i.e. CACP, EACH or EACH-D) of packaged care; duration of package; the presence (or absence) of a cohabiting carer; number and hours of care from non-paid carers; dementia or mental health diagnoses; ethnicity; language spoken; education; previous employment; significant life events/functional change as reported by case manager; case manager; client and care worker positive interaction measured by the 8-item positive relationship subscale of the Caregiver Interaction Scale completed by the family [68]; happiness measured by the 8-item Jovality subscale and 4-item attentiveness subscale of the PANAS-X client self-report (completed once in reference to the time scale of the past two weeks and once in reference to during care worker visits) [62]; severity of cognitive impairment as measured by the Global Deterioration Scale [69]; two items assessing importance to client and family that care workers engage socially with the client; country of birth; years lived in Australia; English proficiency; income; living situation; relationship of family member; relationship between care worker and client as measured by the 4-item Bond subscale of the Working Alliance Inventory Short Form client report [70]	Client/family self-report
		Care plan audit
		Case manager interview
		Client/family interview
Care worker secondary outcome		
Work satisfaction	5-item dedication subscale of the Utrecht Work Engagement Scale [71]	Care worker self-report
Care staff covariates	age; gender; care site; hours of work; duration of employment in current role and in the aged care industry; name of manager; education; ethnicity; language spoken; diversional therapy or lifestyle experience; number of clients/staff supported; relationship between care worker and client as measured by the 4-item Bond subscale of the Working Alliance Inventory Short Form care worker self-report [70]; case manager work satisfaction measured by the 5-item dedication subscale of the Utrecht Work Engagement Scale [71]; country of birth; years lived in Australia; English proficiency; duration of relationship with client; number and duration of care visits to clients over two weeks.	Care staff self-report

*Note*. Translations will be made of all questionnaires, other than those individually noted above where pre-existing translated versions are available.

*Pre-existing translated versions in Spanish and Chinese are available.

^†^Pre-existing translated versions in Spanish, Vietnamese and Arabic are available.

‡Pre-existing translated versions in Arabic and Spanish are available.

##### Translations

Assessments will be conducted in English, Cantonese, Mandarin, Vietnamese, Arabic or Spanish according to the preferred language of participants. Validated translated versions of scales will be used where available (see Table 2). Where they are not available (See Table 2), scales will be translated by accredited translators, then checked for meaning by bilingual research staff with experience in the area.

#### Process evaluation

The process evaluation will assess five components [72,73].

#### Context

Aspects of the environment or organisation that may influence intervention implementation or study outcomes (such as perceived or real barriers, enablers, organisational culture, management support, and attitudes of care staff, clients and families towards LEAP), will be established through interviews with case managers at each evaluation time-point; monthly phone meetings with the LEAP Champions; and bi-annual steering committee meetings.

#### Reach

Care staff attendance at each LEAP training session, and the number of LEAP inductions conducted with new care staff, shall be recorded.

#### Dose delivered

LEAP is a semi-scripted manualised program with set learning objectives, activity aims, activities, handouts and timings. Information about whether each component of the manual is delivered will be recorded. Number of buddy shifts conducted shall also be recorded.

#### Dose received

The extent to which the care staff actively engage with the LEAP program and implement the strategies and activities learnt in training shall be documented by way of case manager interviews, and phone meetings with the LEAP Champion.

A care plan audit will identify whether a SMART social or recreational goal, a vague social or recreational goal, or no goal, has been included in the care plan for each client. The audit will also identify whether or not a personal history sheet documenting clients’ past and present interests, preferences, relationships, roles and so forth is included in the client’s file. Case manager interviews and the care plan audit will collectively inform: 1) how well the goal was (or is being) implemented for each client (0 – not started; 1 – planned; 2 – in progress; 3 – achieved); 2) whether other engagement strategies are being implemented with each client (ranging from 0 - none at all, to 4 - a lot); and 3) reasons if no social or recreational goal has been identified for a client.

Care workers will complete a one week activity diary recording frequency and type of social and recreational activities undertaken with clients.

#### Fidelity

The extent to which LEAP is implemented as planned, shall be established by way of case manager interviews, phone meetings with LEAP Champions, and a review at 12 months of the success of each site-specific implementation plan. In regards to the behaviour of the facilitators, care staff will complete a short written evaluation after each training session.

#### Power

The target sample of 211 clients clustered within 20 case managers will give at least 80% power to detect a small effect size (Cohen’s *d* = 0.2) at a significance level of two sided 0.05, assuming a median intraclass correlation (rho = 0.5) between pre and post intervention measures [74,75]. This power analysis is based on one group comparing pre- and post-measures for the primary outcome measure [75]. As the engagement score is a new measure, we do not know what the correlation structure among the repeated assessments using the instrument will be. Post hoc power analysis will be conducted upon study completion.

#### Data analysis

Piecewise linear mixed effect models will be used as the primary analysis method, with correlations between repeated measurements taken into account. The mean and standard deviation of the pre-intervention slope (-6 to 0 months) and post intervention slope (0 to 12 months) as well as the mean outcome measure at time 0 with 95% Confidence Interval will be estimated. The difference of the two slopes will be tested based on the Z score at the significance level of two-sided 0.05. The model will be fitted with and without covariates. Piecewise least squares analysis will be conducted as supportive analysis. The primary analysis will be performed on the qualified intention to treat (ITT) population of all the clients enrolled in this study. Missing outcome data will be handled by the mixed effect model approach, providing the missing at random (MAR) assumption is met. If appropriate, multiple imputation will be used to generate missing covariate data. Quantitative process evaluation data shall be described. Qualitative process evaluation data shall be described and where appropriate, thematically analysed. Where possible, analyses will also be conducted using process evaluation data as predictors of outcomes.

An estimate of LEAP program costs will be made. This will be based on the costs of both delivering and staff attending the training, time for the LEAP champion, and the cost of any materials or resources required for activities.

## Discussion

LEAP for Life is a pragmatic program with the aim of increasing engagement of aged care clients by aged care staff. The program is designed to be economical to deliver and self-sustaining after the implementation period, and is being evaluated in the real world setting.

The non-controlled design means that we cannot with certainty attribute any observed changes to the LEAP program. However it is unlikely that care worker behaviour with relation to clients would systematically be affected by external factors. The detailed process evaluation will also add confidence in attributing the change to the implementation processes, as well as in understanding the mechanisms and processes involved in changing home care staff behaviour.

## Competing interests

The authors declare that they have no competing interests.

## Authors’ contributions

LFL conceived of the study, obtained funding, led the design and coordination of the study and drafted the manuscript. JB participated in the design and coordination of the study and helped to draft the manuscript. YHJ helped to obtain the funding, participated in the design and provided important review of the manuscript. CC participated in the design and provided important review of the manuscript. MH participated in the design and provided important review of the manuscript. MS participated in the design and provided important review of the manuscript. All authors read and approved the final manuscript.

## Pre-publication history

The pre-publication history for this paper can be accessed here:

http://www.biomedcentral.com/1471-2318/13/124/prepub
